# Rebound Tremor Frequency as a Potential Diagnostic Marker for Delayed Therapy Escape after Thalamic Deep Brain Stimulation for Essential Tremor—Insights from a Cross-Sectional Study

**DOI:** 10.3390/brainsci14070667

**Published:** 2024-06-29

**Authors:** Marvin L. Frommer, Isabelle D. Walz, Franz Aiple, Nils Schröter, Christoph Maurer, Michel Rijntjes, Thomas Prokop, Peter C. Reinacher, Volker A. Coenen, Bastian E. A. Sajonz

**Affiliations:** 1 Department of Stereotactic and Functional Neurosurgery, Medical Center—University of Freiburg, Faculty of Medicine, University of Freiburg, 79106 Freiburg im Breisgau, Germany; 2 Department of Neurology, Medical Center—University of Freiburg, Faculty of Medicine, University of Freiburg, 79106 Freiburg im Breisgau, Germany; 3 Department of Sport and Sport Science, University of Freiburg, 79106 Freiburg im Breisgau, Germany; 4 IT-Department, Neurocenter, Medical Center—University of Freiburg, Faculty of Medicine, University of Freiburg, 79106 Freiburg im Breisgau, Germany; 5 Fraunhofer Institute for Laser Technology (ILT), 52074 Aachen, Germany; 6 Department of Neurosurgery, Kantonsspital St. Gallen, 9000 St. Gallen, Switzerland; 7 Center for Deep Brain Stimulation, University of Freiburg, 79106 Freiburg im Breisgau, Germany

**Keywords:** deep brain stimulation, essential tremor, therapy escape, ataxia, diagnostic marker

## Abstract

Delayed therapy escape (DTE) is frequent after thalamic deep brain stimulation for essential tremor, leading to reduced quality of life, often with ataxic symptoms, and early recognition is challenging. Our goal was to examine whether a low-frequency rebound tremor of the left hand after switching off stimulation is useful as a diagnostic marker for DTE. In this cross-sectional study with additional retrospective analysis, we examined 31 patients with bilateral thalamic DBS ≥ 12 months for essential tremor, using quantitative assessments including video-based motion capture, Fahn–Tolosa–Marin Tremor Rating Scale (FTMTRS), and scale for the assessment and rating of ataxia (SARA). If available, preoperative (preOP) and 12-month postoperative assessments were included in the analysis. Evaluations occurred with DBS activated (ON) and deactivated (OFF). A higher ratio FTMTRS now_ON_/preOP indicated DTE. Preoperative FTMTRS scores were available for 16 patients, including 5 patients with DTE. The receiver operating characteristic analysis found an area under the curve of 0.86 (*p* = 0.024) for identification of DTE by low-frequency rebound tremor (i.e., OFF) on the left. In conclusion, it could serve as a potential diagnostic marker.

## 1. Introduction

Deep brain stimulation (DBS) of the ventral nucleus intermedius (VIM) of the thalamus is effective in treating pharmacotherapy-resistant essential tremor (ET). However, up to 30–40% of patients are affected by loss of benefit over time, termed habituation, tolerance, and late failure [1,2]. This phenomenon lacks a consensus definition; however, Fasano and Helmich proposed the term therapy escape, whereby tremor severity is reduced by DBS by at least 50% for at least 6 months and then subsequently reaches pre-DBS tremor severity within 1 year (early escape) or later (late escape) while on stimulation [3]. This complex phenomenon is associated with ataxic symptoms, suggesting cerebellar dysfunction and impairs quality of life [3]. The pathogenesis of this complex phenomenon is not yet fully understood. Disease-related, stimulation-related, and surgery-related aspects are discussed [1,2,4,5,6,7,8,9]. 

Currently, there is no diagnostic test to determine whether a patient is affected or starting to be affected by DTE. The symptoms and hallmarks develop gradually, which makes it especially difficult to detect early on. As per the definition, we only identify patients with therapy escape once they are severely affected and reach preoperative tremor severity. In a group of patients with DTE, we observed a low-frequency tremor predominantly on the left side upon switching off the stimulation (OFF) [10]. We hypothesize that the frequency of this rebound tremor (i.e., OFF) on the left correlates strongly with DTE and may serve as a diagnostic marker. 

## 2. Methods

### 2.1. Study Design

In this cross-sectional study with additional retrospective analysis, data were collected between August and November 2021 from patients in our outpatient clinic at University Medical Centre Freiburg, Germany. The only inclusion criterion was that participants underwent bilateral thalamic DBS for ET at least 12 months ago. The only exclusion criterion was a constant dependency on walking aids or a wheelchair. This study adhered to the principles of the Helsinki Declaration and received approval from the local ethics committee (623/19). Written informed consent was obtained from all patients. 

### 2.2. Outcome Measures

#### 2.2.1. Patient Demographics and Medical History

The following data were recorded: patient demographics (age, sex), disease specifications (disease onset, disease duration, time since DBS operation), stimulation specifics (daytime adjustments, regular switch off), and handedness. Patients were also asked which hand was more affected by tremor. In addition, a medical chart review was conducted to gather the following information, if available: family history of ET, tremor-related medication, tremor improvement by alcohol, consumption of alcohol and illicit drugs, and smoking status. Furthermore, the following comorbidities potentially interacting with afferent and efferent cerebellar information flow were assessed with medical chart review and by asking the patient: spinal stenosis, pre-existing cerebellar disease, previous chemotherapy, polyneuropathy, and diabetes mellitus (being the most common cause for polyneuropathy).

#### 2.2.2. Fahn–Tolosa–Marin Tremor Rating Scale (FTMTRS)

For clinical assessment of tremors, we used the FTMTRS [11], which is valid and frequently applied (cf. [12]) and facilitated comparability to retrospective data at our center. At the main study visit (“now”), patients were examined with activated stimulation (ON) and directly after switching off (OFF). FTMTRS was the only variable for which retrospective data were gathered in addition to the main visit (“now”). Where available, we collected preoperative (preOP) and 12-month postoperative (12M, [ON]) FTMTRS values. The FTMTRS was acquired unblinded by the following authors: at “now” by M.L.F. and B.E.A.S. All preOP examinations were conducted by T.P., while 12M examinations were mostly conducted by T.P. and some by B.E.A.S. 

#### 2.2.3. Calculated Ratio FTMTRS now_ON_/preOP

Operationalizing a proposed definition of DTE [3], the ratio comparing FTMTRS now_ON_ vs. preoperative indicates the extent of therapy efficacy (<1) and DTE (≥1), respectively. This ratio could only be calculated in patients with available FTMTRS values from both the “preOP” and “now” time points.

#### 2.2.4. Scale for the Assessment and Rating of Ataxia (SARA)

To examine clinical signs of ataxia, we used SARA [13], which is valid and frequently applied (cf. [14]). SARA was assessed in ON and OFF state unblinded by the authors M.L.F. and B.E.A.S. For statistical analysis, we used a modified SARA score without item 6 (nose–finger test measuring tremor), as previously described [15].

#### 2.2.5. Quality of Life in Essential Tremor Questionnaire (QUEST)

The validated German version of the Quality of Life in Essential Tremor Questionnaire (QUEST) was used to assess disease-specific quality of life [16,17]. 

#### 2.2.6. Quantitative Tremor Analysis

Frequency (in Hz) and total power (in milli-gravities^2^/µV^2^) of postural tremor were measured in ON and OFF states as described before [10]. 

#### 2.2.7. Vision-Based Motion Capture

As an objective marker for gait ataxia, mean step lengths of three runs were determined in ON and OFF states with a marker-less vision-based motion capture system, TheCaptury (The Captury GmbH, Saarbrücken, Germany), during a timed up-and-go task, as described before [10].

### 2.3. Statistical Analysis 

Statistical analysis was performed with GraphPad Prism 9 (GraphPad Software, San Diego, CA, USA). The normal distribution of parametric data was determined with the D’Agostino-Pearson test. The distribution of non-parametric variables and parametric variables (mean and standard deviations) was assessed across the whole sample as well as among the patients with and without DTE assigned by their ratio FTMTRS now_ON_/preOP. The two independent groups (patients with vs. without DTE) were compared exploratorily across all outcomes using two-tailed Mann–Whitney U tests for continuous variables and chi-square tests for nominal variables, giving *p*-values for orientation. The test validity of the hypothesized rebound (i.e., OFF) tremor frequency on the left was analyzed by computing receiver operating characteristics (ROC) curves with area under the curve (AUC) and the significance level set to 0.05. To search for additional potential hallmarks of DTE beyond the hypothesized rebound (i.e., OFF) tremor frequency on the left, exploratory correlation analyses were conducted using Pearson’s product–moment correlation coefficient between quantitative tremor features and clinical scores indicating DTE and ataxia. Objective measures (i.e., quantitative tremor features and step length) showing a correlation of bigger effect size than the hypothesized rebound (i.e., OFF) tremor frequency on the left with the ratio FTMTRS now_ON_/preOP were submitted to an exploratory ROC analysis.

## 3. Results

Demographics and clinical data are reported in Table 1. Thirty-one patients were included. When applied to the preoperative condition of all patients diagnosed by board-certified neurologists specialized in movement disorders, consensus criteria [18] reveal the presence of ET in 28 patients and ETplus in three patients. Preoperative FTMTRS values were only available for 16 patients. Gait analysis was only possible in 27 patients because of the unavailability of the motion capture system. Four patients could not perform the test at OFF because of unsteady gait. All data (including the ratio FTMTRS now_ON_/preOP) were normally distributed.

**Figure 1:**
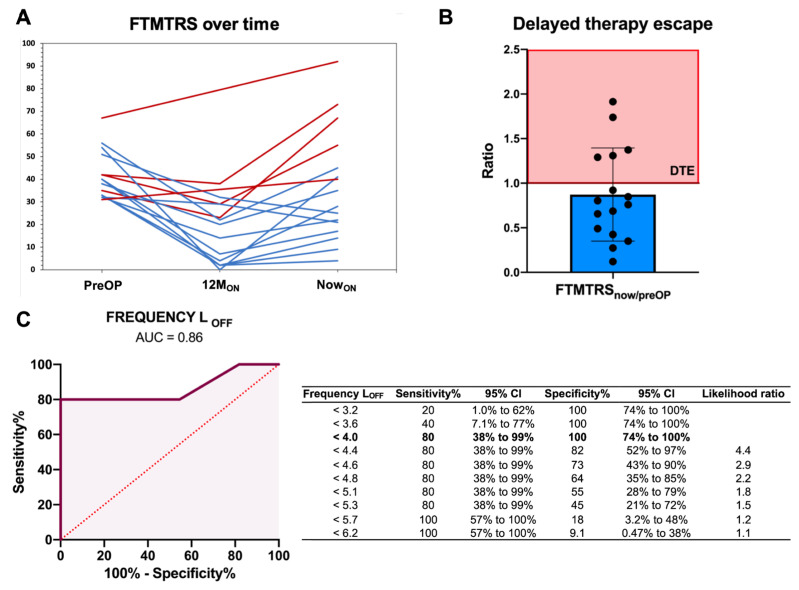
(**A**) Development of FTMTRS values over time. The FTMTRS 12M was unavailable for two patients; however, clinical records indicate satisfactory tremor control at their 12-month postoperative appointment. Note that Now_ON_ is not to scale as intervals between 12M_ON_ and Now_ON_ vary across patients (red lines: patients with DTE; blue lines: patients without DTE). (**B**) Distribution of ratio FTMTRS now_ON_/preOP across patients. (**C**) ROC curve depicting AUC for identification of DTE by left tremor frequency at OFF with adjacent table specifying the ideal cut-off value. Abbreviations: 12M, at 12 months postoperatively; AUC, area under the curve; DTE, delayed therapy escape; FTMTRS, Fahn—Tolosa—Marín tremor rating scale; Now, at the main study point; OFF, stimulation off state; ON, stimulation on state; preOP, preoperatively.

Among patients with available ratio FTMTRS now_ON_/preOP, we identified five patients to have a ratio > 1, indicating DTE (Figure 1A,B). All patients scored lower on the FTMTRS at 12M_ON_ compared to preoperatively, but to some extent, all patients experienced a worsening of their tremor control over time until the “now” time point (Figure 1A). The distribution of variables across patients with and without DTE and between-group comparisons is provided in Supplementary Table S1.

In the ROC analysis for tremor frequency LOFF and DTE, the AUC was 0.86 (*p* = 0.024, 95% CI [0.61, 1.0]) (Figure 1C). A cut-off value of 4 Hz or less was associated with a sensitivity of 80% and a specificity of 100% (Figure 1C). The exploratory correlation analysis revealed that tremor frequency on the left side at ON (compared to OFF) showed an association of bigger effect size with the ratio FTMTRS now_ON_/preOP (Supplementary Figure S1), and the exploratory ROC analysis found a similar diagnostic accuracy (Supplementary Figure S2).

## 4. Discussion

**Figure 2:**
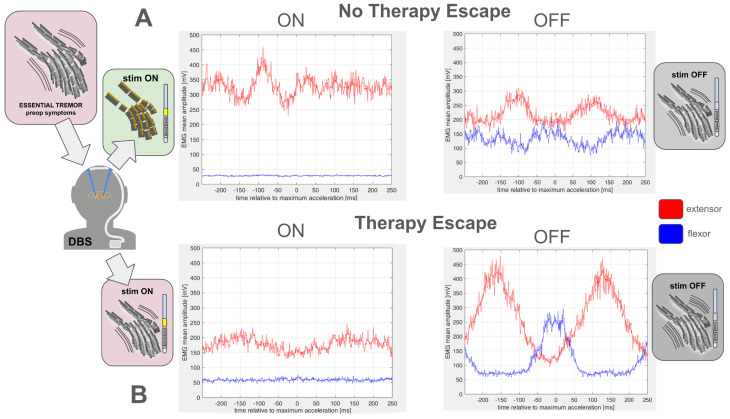
Exemplary comparison of mean EMG signals of a patient without (**A**) and with therapy escape (**B**). Two channels of EMG signals (flexor, extensor) were acquired along with the acceleration signal (ACC) for a duration of 30 s using a sampling rate of 1000 Hz. For this illustration, the left-hand signals were used, as usually, the tremor was stronger in the left than in the right hand. ACC was bandpass filtered to the range 2–10 Hz. Single tremor oscillation cycles were defined as the time from one zero crossing from negative to positive ACC values to the next zero crossing from negative to positive ACC values. Times of the maximum ACC value in each cycle were obtained (tmax), resulting in, e.g., 158 time values for a 30 s recording. EMG was high pass filtered with low-frequency cut-off at 2 Hz, then rectified (absolute value of EMG). The EMG segments corresponding to the time span of 250 ms before to 250 ms after each maximum acceleration time (tmax) were averaged and plotted (blue line: flexor; red line: extensor). An illustration of this averaging process is given in Supplementary Figure S3.

In our cohort, a low-frequent postural tremor at OFF on the left side with 4 Hz or less effectively identified DTE with a sensitivity of 80% and a specificity of 100%. Tremor frequency is commonly used for the classification of tremor syndromes [18,19]. Specifically, frequencies below 5 Hz are linked to intention tremor and Holmes tremor [18]. ET, on the other hand, generally exhibits higher frequencies, and occurrences below 4 Hz rule out ET and point to a rubral or cerebellar tremor [20]. Tremor frequency may decrease in the course of ET but never falls below 4 Hz [20]. However, time since DBS and disease duration did not differ between patients with DTE and patients without DTE in our sample (Supplementary Table S1). Hence, clinically, the left-sided tremor in patients with DTE can be classified as an intention tremor syndrome, combined with other signs of ataxia (cf. Supplementary Figure S1), superimposed on ET. As computational models suggest that Vim-DBS leads to an increase in tremor frequency [21], our findings possibly arise from long-term neuroplasticity in response to DBS that may form the pathophysiological process underlying DTE. Using an accelerometer and EMG, we found a significantly higher mean amplitude at OFF in patients with DTE (Supplementary Table S1, Figure 2), correlating with higher total power and lower tremor frequency in patients with DTE at OFF. Moreover, curves for extensors and flexors overlap, suggesting that tremor in DTE less coordinated. This may be an underlying aspect of the ataxic clinical presentation of DTE.

In all patients affected by DTE, tremors and ataxia were more pronounced on the left and hence non-dominant side. The tremor frequency on the left rather than the right side is especially correlated with DTE, FTMTRS ON, and SARA ON (Supplementary Figure S1). Thus, a new manifestation of a non-stimulation-induced left-sided hemiataxia could be an alternative explanation deemed, however, less likely by us considering the distribution of potential causes across patients with and without DTE (Supplementary Table S1) and the exacerbation elicited by switching off the stimulation. We have recently demonstrated that electrode location with disbalanced recruitment favoring uncrossed over crossed cerebellothalamic pathways in the right thalamus by Vim-DBS is predictive of DTE with predominantly left-sided symptomatology and may represent an underlying mechanism for DTE [22]. In addition, a variety of other aspects are associated with DTE and may play a role in its evolution, i.e., disease progression, pre-existing cerebellar disease, demyelinating neuropathy, high stimulation pulse width, antidromic stimulation effects on the cerebellum, and shorter disease duration at surgery and older age at surgery [1,2,4,5,6,7,8,9,10].

Measuring tremor frequency at OFF is a simple and quick diagnostic method, necessitating no additional waiting time after switching off, as the rebound effect typically diminishes within 30–60 min [23]. It can be performed with both laboratory-grade accelerometers or accelerometers in consumer products (smartphones, smartwatches, etc.) [24], and it can easily be incorporated into regular outpatient visits or even virtual appointments. Utilizing it as an indicator of therapy escape would facilitate the monitoring of this phenomenon. Its gradual onset makes it challenging to detect until it reaches an advanced stage, emphasizing the importance of an early detection method. Continuously lowering frequencies approaching 4 Hz could be indicative. However, we only show that low frequent tremor correlates with established DTE. A longitudinal prospective cohort study monitoring frequency over time and its ability to predict DTE is needed. The ability to continuously monitor progression would allow for the implementation of early therapeutic measures like optimization of stimulation parameters [25], including pulse width reduction [26,27], pausing stimulation [8,28], and revision surgery.

Complimentary to our previous findings [10], we further found tremor frequency at ON on the left side to be effective in identifying DTE, too, with a slightly higher AUC. Even at ON, patients with DTE showed frequencies of less than 4 Hz. However, when selecting a cut-off value, the frequency at ON is less indicative, with no combination of sensitivity and specificity as high as when compared to the frequency at OFF (Supplementary Figure S2). Nevertheless, the frequency at ON on the left seems to be an efficient marker for identifying DTE and should be considered in future diagnostic workups of this phenomenon alongside tremor frequency at OFF on the left.

Limitations of this study include the small sample size, missing data, and reliance on the retrospective and unblinded collection of FTMTRS values to identify DTE. Here, we adopted Fasano and Helmich’s proposed definition of DTE; however, there is no universally accepted definition, which makes standardization and comparability between studies difficult [3]. Without left-handers among patients with therapy escape, given the aforementioned issues, generalisability is limited. Although our results denote an effect of hemispheric dominance, we did not employ neuropsychological inventories or fMRI paradigms for verification and cannot exclude right hemispheric dominance in patients identifying themselves as right-handed and vice versa. However, we expect that this would reduce the power of our model. As DTE appears to be associated with signs of ataxia in our sample (especially at OFF, cf. Supplementary Figure S1), we cannot exclude that DTE is a separate process preceding ataxia. In this case, however, our findings could still be regarded as a surrogate marker of DTE.

In conclusion, we suggest a low-frequent rebound tremor of 4 Hz or less on the left side as a potential diagnostic tool to detect delayed therapy escape in ET patients with VIM-DBS. Integrating this quick and low-cost tool into the routine outpatient setting might enable early detection and, hence, the opportunity for early countermeasures. Bigger, preferably multi-center, studies with blinded examinations are needed to improve validity and further explore suitability as a diagnostic marker.

## Figures and Tables

**Table 1:** Patient demographics and basic clinical data.

Variable	Data Distribution
	*n*
Sex (Male/Female)	18:13
Handedness (Right/Left)	29:2
Hand more affected by ET (Right/Left)	11:20
Daytime stimulation adjustments (Yes/No)	15:16
Overnight stimulation switch off (Yes/No/Lower)	9:21:1
Family history of ET (Yes/No, *n* = 21)	17:4
Tremor improved by alcohol? (Yes/No, *n* = 17)	12:5
Frequent consumption of alcohol? (Yes/No)	2:29
Smoking (Yes/No)	6:25
Consumption of illicit drugs (Yes/No)	0:31
Tremor-related medication? (Yes/No)	10:21
Comorbidities	
Spinal stenosis (Yes/No)	1:30
Pre-existing Cerebellar disease (Yes/No)	0:31
Previous Chemotherapy (Yes/No)	1:30
Polyneuropathy (Yes/No)	8:23
Diabetes mellitus (Yes/No)	12:19
	Mean ± SD (range)
Age	72.06 ± 8.6 (52–85)
Disease onset (years)	31.06 ± 18.6 (6–67)
Disease duration (years)	39.97 ± 18.4 (4–65)
Time since DBS implantation (years)	6.4 ± 4.4 (1.5-–18)
Medication	
Propranolol (mg, *n* = 5) ^1^	88 ± 43 (40–160)
Primidone (mg, *n* = 3) ^1^	437.5 ± 187.5 (250–625)
Gabapentin (mg, *n* = 1)	1600
Topiramate (mg, *n* = 1)	50
Clonazepam (mg, *n* = 1)	1.5
FTMTRS preOP (*n* = 16)	41 ± 11 (31–67)
FTMRS 12M ON (*n* = 14)	16 ± 13 (0–38)
FTMTRS ON	36 ± 21 (4–92)
FTMTRS OFF	65 ± 22 (13–112)
Ratio FTMTRS now_ON_/preOP (*n* = 16)	0.87 ± 0.52 (0.12–1.9)
SARA without item 6 ON	7.4 ± 4.3 (1–16)
SARA without item 6 OFF	6.5 ± 4.5 (0–18)
Step Length ON (meters) (*n* = 27) ^2^	0.41 ± 0.09 (0.25–0.57)
Step Length OFF (meters) (*n* = 23) ^3^	0.44 ± 0.07 (0.30–0.53)
Postural Tremor Frequency ON Mean (Hz)	6.0 ± 1.2 (3.4–8.4)
Postural Tremor Frequency OFF Mean (Hz)	4.9 ± 1.1 (3.3–7.8)
Postural Tremor Frequency OFF Right (Hz)	5.0 ± 1.3 (3.5–8.2)
Postural Tremor Frequency OFF Left (Hz)	4.8 ± 1.1 (3.0–7.4)
QUEST	
Health Overall	70 ± 20 (30–100)
Quality of Life Overall	71 ± 20 (31–100)
Communication	19.4 ± 23.1 (0–75)
Work and Finances	14.8 ± 24.1 (0–100)
Hobbies	38.2 ± 34.4 (0–91.7)
Physical	51.5 ± 35.3 (0–100)
Psychosocial	22.8 ± 22.5 (0–86.1)

*Note:* If not indicated otherwise, data were acquired at the main study visit (“now”). Where there are no numbers in parentheses in the variable column, data from all 31 patients were available. ^1^ One patient took propranolol and primidone. ^2^ A total of 4 participants could not perform the gait analysis due to the unavailability of motion capture systems. ^3^ A total of 4 participants could not perform the gait analysis because of a too-unsteady gait at OFF. Abbreviations: ET, essential tremor; SD, standard deviation; DBS, deep brain stimulation; FTMTRS, Fahn–Tolosa–Marin Tremor Rating Scale; 12M, at 12 months postoperatively; OFF, stimulation OFF state; ON, stimulation ON state; preOP, preoperative; SARA, scale for the assessment and rating of ataxia; QUEST, Quality of Life in Essential Tremor Questionnaire.

## Data Availability

Due to ethical restrictions, the data presented in this study are only available upon reasonable request from the corresponding author and after approval from the institutional review board.

## Supplementary Materials

The following supporting information can be downloaded at: https://www.mdpi.com/article/10.3390/brainsci14070667/s1, Supplementary Table S1: Comparison between patients with therapy escape and patients without therapy escape. Supplementary Figure S1: Correlation of quantitative tremor features and clinical scores indicating therapy escape and ataxia. Supplementary Figure S2: Receiver operating curve for postural tremor frequency at ON on the left side to predict DTE. Supplementary Figure S3: Exemplary illustration of the averaging process of EMG segments of left flexor and extensor corresponding to the time span of 250 ms before to 250 ms after each maximum acceleration time of the patient with therapy escape with switched-off stimulation. Single (colored) EMG segments in this figure are added successively with a 4 mV separation on the y-axis to avoid overlap. The black graph gives the mean of all EMG segments and is magnified by the factor 10 to better point out the overall effect. The scale of the y-axis corresponds to the black mean graph. The resulting (black) graphs of the means are depicted together in Figure 2B on the right.

## Abbreviations

AUC	Area under the curve
DBS	Deep brain stimulation
DTE	Delayed therapy escape
FTMTRS	Fahn–Tolosa–Marin Tremor Rating Scale
EMG	Electromyography
ET	Essential tremor
ON	Activated stimulation
OFF	Deactivated stimulation
preOP	preoperative
QUEST	Quality of Life in Essential Tremor Questionnaire
ROC	Receiver operating curve
SARA	Scale for the assessment and rating of ataxia
12M	12 months postoperatively

## References

[1] Chiu S.Y., Nozile-Firth K., Klassen B.T., Adams A., Lee K., Van Gompel J.J., Hassan A. (2020). Ataxia and tolerance after thalamic deep brain stimulation for essential tremor. Park. Relat. Disord..

[2] Pilitsis J.G., Metman L.V., Toleikis J.R., Hughes L.E., Sani S.B., Bakay R.A.E. (2008). Factors involved in long-term efficacy of deep brain stimulation of the thalamus for essential tremor. J. Neurosurg..

[3] Fasano A., Helmich R.C. (2019). Tremor habituation to deep brain stimulation: Underlying mechanisms and solutions. Mov. Disord..

[4] Anthofer J.M., Steib K., Lange M., Rothenfusser E., Fellner C., Brawanski A., Schlaier J. (2017). Distance between Active Electrode Contacts and Dentatorubrothalamic Tract in Patients with Habituation of Stimulation Effect of Deep Brain Stimulation in Essential Tremor. J. Neurol. Surg. A Cent. Eur. Neurosurg..

[5] Favilla C.G., Ullman D., Wagle Shukla A., Foote K.D., Jacobson C.E., Okun M.S. (2012). Worsening essential tremor following deep brain stimulation: Disease progression versus tolerance. Brain.

[6] Merchant S.H., Kuo S.-H., Yu Q., Winfield L., McKhann G., Sheth S., Pullman S.L., Ford B. (2018). Objective predictors of “early tolerance” to ventral intermediate nucleus of thalamus deep brain stimulation in essential tremor patients. Clin. Neurophysiol..

[7] Patel N., Ondo W., Jimenez-Shahed J. (2014). Habituation and rebound to thalamic deep brain stimulation in long-term management of tremor associated with demyelinating neuropathy. Int. J. Neurosci..

[8] Reich M.M., Brumberg J., Pozzi N.G., Marotta G., Roothans J., Åström M., Musacchio T., Lopiano L., Lanotte M., Lehrke R. (2016). Progressive gait ataxia following deep brain stimulation for essential tremor: Adverse effect or lack of efficacy?. Brain.

[9] Shih L.C., LaFaver K., Lim C., Papavassiliou E., Tarsy D. (2013). Loss of benefit in VIM thalamic deep brain stimulation (DBS) for essential tremor (ET): How prevalent is it?. Park. Relat. Disord..

[10] Sajonz B.E.A., Frommer M.L., Walz I.D., Reisert M., Maurer C., Rijntjes M., Piroth T., Schröter N., Jenkner C., Reinacher P.C. (2022). Unravelling delayed therapy escape after thalamic deep brain stimulation for essential tremor?—Additional clinical and neuroimaging evidence. Neuroimage Clin..

[11] Fahn S., Tolosa E., Marin C., Jankovic J., Tolosa E. (1993). Clinical Rating Scale for Tremor. Parkinson’s Disease and Movement Disorders.

[12] Elble R.J., Ondo W. (2022). Tremor rating scales and laboratory tools for assessing tremor. J. Neurol. Sci..

[13] Schmitz-Hübsch T., du Montcel S.T., Baliko L., Berciano J., Boesch S., Depondt C., Giunti P., Globas C., Infante J., Kang J.S. (2006). Scale for the assessment and rating of ataxia: Development of a new clinical scale. Neurology.

[14] Grobe-Einsler M., Amin A.T., Faber J., Völkel H., Synofzik M., Klockgether T. (2024). Scale for the assessment and rating of ataxia (SARA): Development of a training tool and certification program. Cerebellum.

[15] Roque D.A., Hadar E., Zhang Y., Zou F., Murrow R. (2022). Reducing Ataxic Side Effects from Ventral Intermediate Nucleus of the Thalamus Deep Brain Stimulation Implantation in Essential Tremor: Potential Advantages of Directional Stimulation. Stereotact. Funct. Neurosurg..

[16] Tröster A.I., Pahwa R., Fields J.A., Tanner C.M., Lyons K.E. (2005). Quality of life in Essential Tremor Questionnaire (QUEST): Development and initial validation. Park. Relat. Disord..

[17] Hopfner F., Nebel A., Lyons K.E., Tröster A.I., Kuhlenbäumer G., Deuschl G., Martinez-Martin P. (2016). Validation of the QUEST for German-speaking countries. Int. J. Neurosci..

[18] Bhatia K.P., Bain P., Bajaj N., Elble R.J., Hallett M., Louis E.D., Raethjen J., Stamelou M., Testa C.M., Deuschl G. (2018). Consensus Statement on the classification of tremors. from the task force on tremor of the International Parkinson and Movement Disorder Society. Mov. Disord..

[19] Deuschl G., Bain P., Brin M. (1998). Consensus statement of the Movement Disorder Society on Tremor. Ad Hoc Scientific Committee. Mov. Disord..

[20] Deuschl G., Krack P., Lauk M., Timmer J. (1996). Clinical neurophysiology of tremor. J. Clin. Neurophysiol..

[21] Zhang X., Santaniello S. (2019). Role of cerebellar GABAergic dysfunctions in the origins of essential tremor. Proc. Natl. Acad. Sci. USA.

[22] Sajonz B.E.A., Frommer M.L., Reisert M., Blazhenets G., Schröter N., Rau A., Prokop T., Reinacher P.C., Rijntjes M., Urbach H. (2024). Disbalanced recruitment of crossed and uncrossed cerebello-thalamic pathways during deep brain stimulation is predictive of delayed therapy escape in essential tremor. Neuroimage Clin..

[23] Paschen S., Forstenpointner J., Becktepe J., Heinzel S., Hellriegel H., Witt K., Helmers A.-K., Deuschl G. (2019). Long-term efficacy of deep brain stimulation for essential tremor: An observer-blinded study. Neurology.

[24] van Brummelen E.M.J., Ziagkos D., de Boon W.M.I., Hart E.P., Doll R.J., Huttunen T., Kolehmainen P., Groeneveld G.J. (2020). Quantification of tremor using consumer product accelerometry is feasible in patients with essential tremor and Parkinson’s disease: A comparative study. J. Clin. Mov. Disord..

[25] Contarino M.F., van Coller R., Mosch A., van der Gaag N.A., Hoffmann C.F. (2017). Clinical approach to delayed-onset cerebellar impairment following deep brain stimulation for tremor. Brain.

[26] Soh D., Lozano A.M., Fasano A. (2019). Hybrid deep brain stimulation system to manage stimulation-induced side effects in essential tremor patients. Park. Relat. Disord..

[27] Kroneberg D., Ewert S., Meyer A.-C., Kühn A.A. (2019). Shorter pulse width reduces gait disturbances following deep brain stimulation for essential tremor. J. Neurol. Neurosurg. Psychiatr..

[28] Garcia Ruiz P., Muñiz de Igneson J., Lopez Ferro O., Martin C., Magariños Ascone C. (2001). Deep brain stimulation holidays in essential tremor. J. Neurol..

