# Supplementary material for: Rebound Tremor Frequency as a Potential Diagnostic Marker for Delayed Therapy Escape after Thalamic Deep Brain Stimulation for Essential Tremor—Insights from a Cross-Sectional Study
Source: Brain Sci. 2024 Jun 29;14(7):667. doi: 10.3390/brainsci14070667 (PMC11274735; doi:10.3390/brainsci14070667)
Supplement: Supplementary file 1 [file brainsci-14-00667-s001.zip › brainsci-3042808-supplementary.pdf]

Supplementary Material

Supplementary Table S1. Comparison between patients with therapy escape and patients without therapy escape

**Variable**	**Control**	**Escaper**	**P value**
	n	*n*	
Number of patients	11	5	
Sex (Male:Female)	7:4	2:3	0.377
Handedness (Right:Left)	9:2	5:0	0.308
Hand more affected (Right:Left)	6:5	0:5	**0.036**
Overnight stimulation switch off switch off (Yes:No)	5:6	5:0	**0.036**
Familiy history of ET (Yes:No)	6:1	4:1	0.793
Tremor improved by alcohol? (Yes:No)	3:2	3:1	0.635
Frequent consumption of alcohol? (Yes:No)	1:10	0:5	0.486
Smoking (Yes:No)	2:9	1:4	0.931
Consumption of illicit drugs (Yes: No)	0:11	0:5	–
Tremor related medication? (Yes:No)	3:9	2:3	0.536
Comorbidities			
Diabetes mellitus (Yes:No)	6:5	2:3	0.589
Polyneuropathy (Yes:No)	4:7	1:4	0.512
Spinal stenosis (Yes:No)	0:11	0:5	–
Previous chemotherapy (Yes:No)	0:11	0:5	–
Preexisting cerebellar disease (Yes:No)	0:11	0:5	–
	Mean ± SD	*Mean ± SD*	
Age	68.64 ± 9.68	73.60 ± 8.05	0.391
Disease onset (years)	31.91 ± 25.2	44.60 ± 8.96	0.425
Disease duration (years)	34.46 ± 21.9	28.40 ± 15.7	0.599
Time since DBS implantation (years)	3.80 ± 1.39	3.67 ± 1.65	0.681
Tremor related Medication			
Propranolol (mg)	80 (n=1)	80 ± 0 (n=2)	–
Primidone (mg)	625 (n=1)	437.5 (n=1)	–
Topiramate (mg)	50 (n=1)	–	–
**QUEST**			
Health Overall	75.0 ± 20.6	66.0 ± 18.2	0.295
Quality of Life Overall	80.4 ± 15.9	55.0 ± 16.6	**0.017**

Communication	14.4 ± 23.9	21.7 ± 22.7	0.514
Work and Finances	10.9 ± 17.5	14.2 ± 17.1	0.580
Hobbies	21.2 ± 31.0	65.0 ± 31.4	**0.014**
Physical	33.1 ± 31.2	78.9 ± 17.7	**0.009**
Psychosocial	14.6 ± 19.4	20.0 ± 12. 0	0.259

Supplementary Table S1. (continued)

**FTMTRS**			
Preoperative	40.2 ± 9.2	43.4 ± 14.0	0.641
12M ON	12.2 ± 11.8	30.0 ± 7.5	**0.041**
ON	23.7 ± 12.9	65.4 ± 19.5	**0.002**
OFF	60.2 ± 24.7	80.4 ± 21.7	0.095
Ratio FTMTRS ON/preoperative	0.58 ± 0.26	1.52 ± 0.28	**<.001**
**SARA (no item 6)**			
ON	6.1 ± 4.6	10.0 ± 5.3	0.186
OFF	4.8 ± 3.9	10.0 ± 6.7	0.137
**Step Length**			
ON	0.44 ± 0.09	0.34 ± 0.11	0.095
OFF	0.47 ± 0.07	0.48 ± 0.00	0.711
**Tremor Frequency**			
Mean			
ON	6.22 ± 1.3	4.89 ± 1.0	**0.041**
OFF	5.08 ± 0.86	3.97 ± 0.74	**0.028**
Right			
ON	6.26 ± 1.57	5.8 ± 1.51	0.603
OFF	5.07 ± 1.12	4.12 ± 0.63	0.109
Left			
ON	6.18 ± 1.19	3.98 ± 0.94	**0.006**
OFF	5.09 ± 0.69	3.82 ± 0.88	**0.021**
**Tremor Power**			
Right			
ON	0.232 ± 0.69	0.264 ± 0.47	0.154
OFF	87.74 ± 186	63.94 ± 129	0.827
Left			
ON	1.59 ± 4.30	173.6 ± 297	**0.021**
OFF	14.76 ± 24.0	428.9 ± 343	**0.013**

Note: P values in bold font are considered statistically significant.

Abbreviations: SD, standard deviation; ns, not significant; DBS, deep brain stimulation; FTMTRS, Fahn-Tolosa Marin Tremor Rating Scale; 12M ON, at 12 months postoperatively and ON; SARA, scale for the assessment and rating of ataxia; QUEST, Quality of Life in Essential Tremor Questionnaire.

Supplementary Figure S1. Correlation of quantitative tremor features and clinical scores indicating therapy escape and ataxia: The upper heat map reports Pearson's product moment correlation coefficients (r). The table underneath shows the corresponding p-values for orientation.

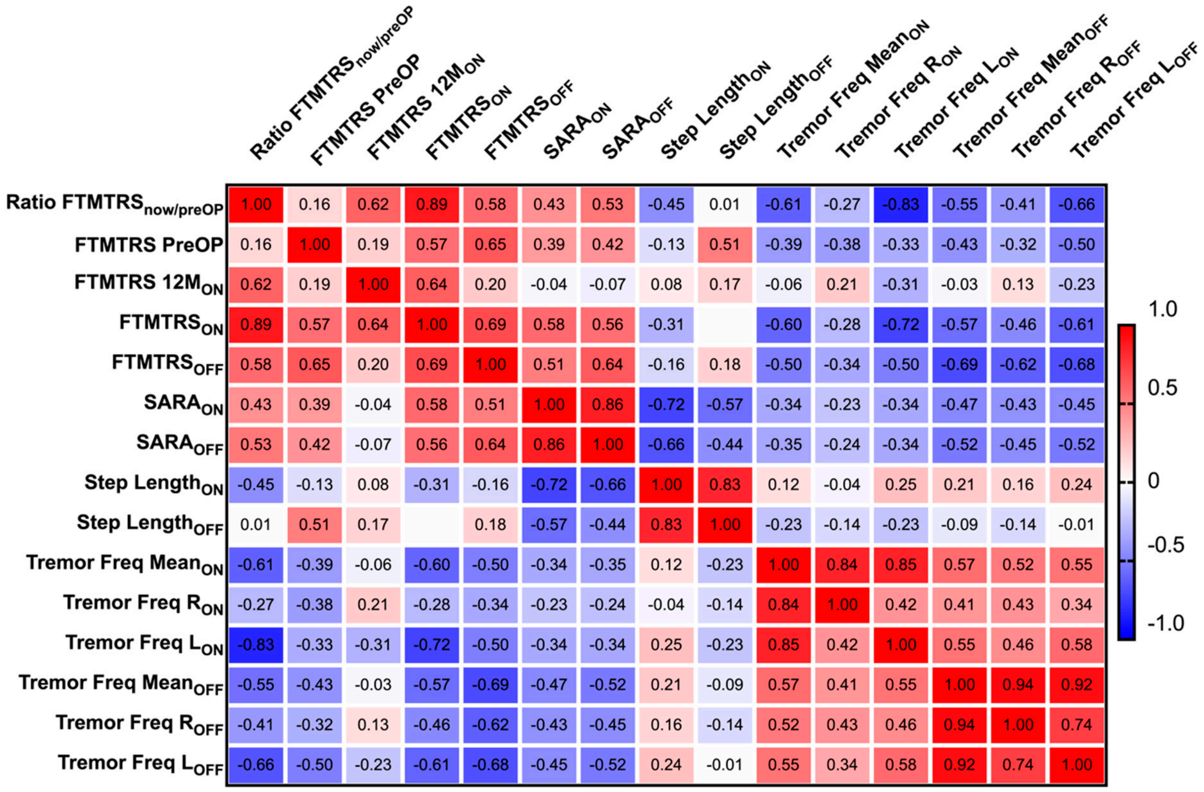

	Ratio FTMTRS$_{now/preOP}$	FTMTRS PreOP	FTMTRS 12M$_{ON}$	FTMTRS$_{ON}$	FTMTRS$_{OFF}$	SARA$_{ON}$	SARA$_{OFF}$	Step Length$_{ON}$	Step Length$_{OFF}$	Tremor Freq Mean$_{ON}$	Tremor Freq R$_{ON}$	Tremor Freq L$_{ON}$	Tremor Freq Mean$_{OFF}$	Tremor Freq R$_{OFF}$	Tremor Freq L$_{OFF}$
Ratio FTMTRS$_{now/preOP}$	0.561	0.019	<.0001	0.019	0.094	0.034	0.110	0.982	0.012	0.305	<.0001	0.026	0.117	0.005	0.005
FTMTRS PreOP	0.561		0.517	0.022	0.007	0.133	0.103	0.656	0.129	0.133	0.145	0.219	0.100	0.232	0.047
FTMTRS 12M$_{ON}$	0.019	0.517		0.014	0.484	0.894	0.823	0.804	0.656	0.840	0.478	0.282	0.913	0.663	0.439
FTMTRS$_{ON}$	<.0001	0.022	0.014		<.0001	<.001	0.001	0.121	0.995	<.001	0.132	<.0001	0.001	0.009	<.001
FTMTRS$_{OFF}$	0.019	0.007	0.484	<.0001		0.004	<.0001	0.434	0.413	<.001	0.062	0.004	<.001	<.001	<.0001
SARA$_{ON}$	0.094	0.133	0.894	<.001	0.004		<.0001	<.0001	0.004	0.061	0.207	0.062	0.008	0.016	0.011
SARA$_{OFF}$	0.034	0.103	0.823	0.001	<.0001	<.0001		<.001	0.037	0.054	0.186	0.059	0.003	0.011	0.003
Step Length$_{ON}$	0.110	0.656	0.804	0.121	0.434	<.0001	<.001		<.0001	0.541	0.834	0.213	0.294	0.429	0.230
Step Length$_{OFF}$	0.982	0.129	0.656	0.995	0.413	0.004	0.037	<.0001		0.287	0.513	0.285	0.683	0.520	0.953
Tremor Freq Mean$_{ON}$	0.012	0.133	0.840	<.001	0.004	0.061	0.054	0.541	0.287		<.0001	<.0001	0.001	0.002	0.001
Tremor Freq R$_{ON}$	0.305	0.145	0.478	0.132	0.062	0.207	0.186	0.834	0.513	<.0001		0.019	0.021	0.017	0.061
Tremor Freq L$_{ON}$	<.0001	0.219	0.282	<.0001	0.004	0.062	0.059	0.213	0.285	<.0001	0.019		0.001	0.010	0.001
Tremor Freq Mean$_{OFF}$	0.026	0.100	0.913	<.001	<.0001	0.008	0.003	0.294	0.683	0.001	0.021	0.001		<.0001	<.0001
Tremor Freq R$_{OFF}$	0.117	0.232	0.663	0.009	<.001	0.016	0.011	0.429	0.520	0.002	0.017	0.010	<.0001		<.0001
Tremor Freq L$_{OFF}$	0.005	0.047	0.439	<.001	<.0001	0.011	0.003	0.230	0.953	0.001	0.061	0.001	<.0001	<.0001	

Abbreviations: 12M, at 12 months postoperatively; Freq, frequency; FTMTRS, Fahn-Tolosa-Marín tremor rating scale; OFF, switched off stimulation, ON, switched on stimulation, preOP, preoperative; SARA, Scale for the assessment and rating of ataxia. Note: if not indicated otherwise (e.g. preOP or 12M) variables relate to the main time point of the study ("now").

Supplementary Figure S2. Receiver operating curve for postural tremor frequency at ON on the left side to predict DTE.

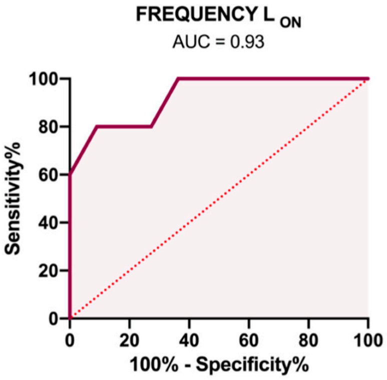

Frequency L_{OFF}	Sensitivity%	95% CI	Specificity%	95% CI	Likelihood ratio
< 3.2	20	1.0% to 62%	100	74% to 100%	
< 3.5	40	7.1% to 77%	100	74% to 100%	
**< 4.1**	**60**	**23% to 93%**	**100**	**74% to 100%**	
**< 4.7**	**80**	**38% to 99%**	**91**	**62% to 100%**	**8.8**
< 5.1	80	38% to 99%	82	52% to 97%	4.4
< 5.3	80	38% to 99%	73	43% to 90%	2.9
< 5.6	100	57% to 100%	64	35% to 85%	2.8
< 5.9	100	57% to 100%	55	28% to 79%	2.2
< 6.3	100	57% to 100%	45	21% to 72%	1.8
< 6.6	100	57% to 100%	36	15% to 65%	1.6
< 7.0	100	57% to 100%	27	9.7% to 57%	1.4
< 7.5	100	57% to 100%	18	3.2% to 48%	1.2
< 7.9	100	57% to 100%	9.1	0.47% to 38%	1.1

Abbreviations: DTE, delayed therapy escape; AUC, area under the curve. The AUC for postural tremor at ON on the left side is 0.93 (p = 0.0078).

Supplementary Figure S3. Exemplary illustration of the averaging process of EMG segments of left flexor and extensor.

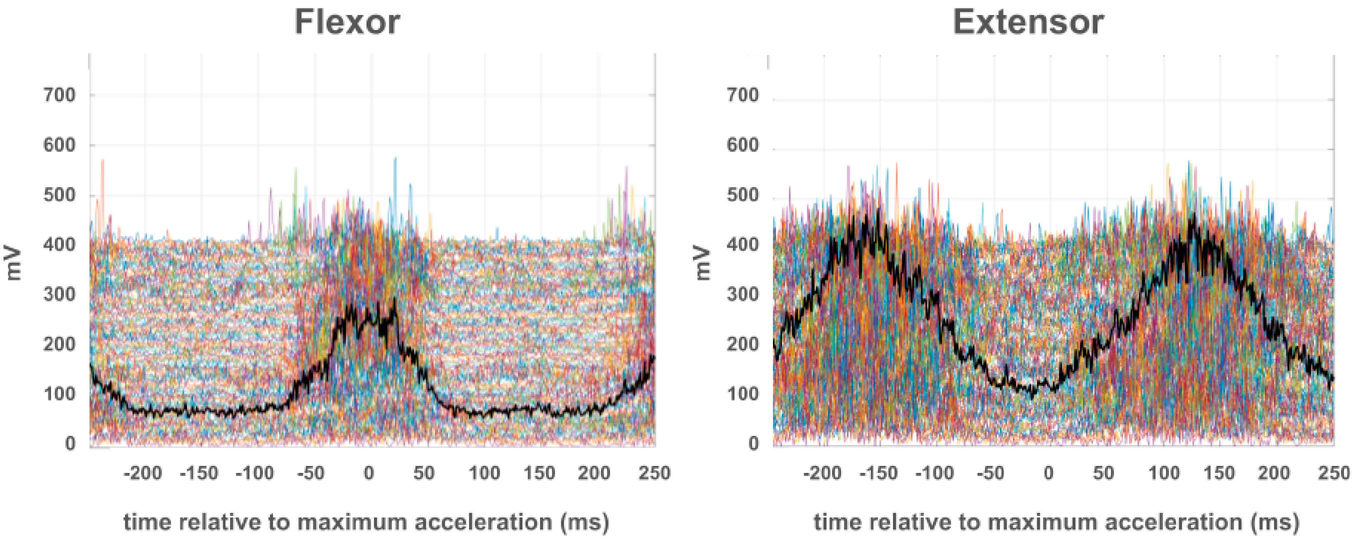

Exemplary illustration of the averaging process of EMG segments of left flexor and extensor corresponding to the time span of 250 ms before to 250 ms after each maximum acceleration time of the patient with therapy escape with switched off stimulation. Single (colored) EMG segments in this figure are added successively with a 4 mV separation on the y-axis to avoid overlap. The black graph gives the mean of all EMG segments and is magnified by the factor 10 to better point out the overall effect. The scale of the y-axis corresponds to the black mean graph. The resulting (black) graphs of the means are depicted together in Figure 2B on the right.
